# Supplementary material for: Tuning interfacial Dzyaloshinskii-Moriya interactions in thin amorphous ferrimagnetic alloys
Source: Sci Rep. 2020 May 4;10:7447. doi: 10.1038/s41598-020-64427-0 (PMC7198596; doi:10.1038/s41598-020-64427-0)
Supplement: Supplementary file 1 — Supplementary information. [file 41598_2020_64427_MOESM1_ESM.pdf]

Supplementary Materials

Tuning interfacial Dzyaloshinskii-Moriya interactions in thin amorphous ferrimagnetic alloys

Y. Quessab,[1,*] J.-W. Xu,[1] C. T. Ma,[2] W. Zhou,[2] G. A. Riley,[3,4] J. M. Shaw,[3] H. T. Nembach,[3,5] S. J. Poon,[2] and A. D. Kent[1]

[1]Center for Quantum Phenomena, Department of Physics, New York University, New York, New York 10003, USA
[2]Department of Physics, University of Virginia, Charlottesville, Virginia 22904, USA
[3]Quantum Electromagnetics Division, National Institute of Standards and Technology, Boulder, Colorado 80305, USA
[4]Center for Memory and Recording Research, University of California San Diego, La Jolla, CA92093, USA
[5]JILA, University of Colorado, Boulder, Colorado 80305, USA

1. Magnetic characterization

The magnetic properties of the CoGd films were measured by vibrating sample magnetometry before BLS studies of the DMI. The room temperature saturation magnetization (M_S), coercive field ($\mu_0 H_C$) and magnetic compensation temperature (T_M) are summarized in the tables below as a function of the CoGd thickness.

Table 1: Summary of the room temperature magnetic properties of Pt/CoGd(*t* nm)/(Pt or W) measured by VSM as a function of magnetic thickness *t* (nm).

**Pt cap layer**			
t (nm)	M_S (kA m^{-1})	$\mu_0 H_C$ (mT)	T_M (K)
5	160	11.0	125 – 150
10	156	0.6	125
15	125	0.6	150

**W cap layer**			
t (nm)	M_S (kA m^{-1})	$\mu_0 H_C$ (mT)	T_M (K)
5	145	11.0	125 – 150
8	144	11.0	150
10	146	0.6	125
15	140	0.6	150

*yassine.quessab@nyu.edu

2. Evidence of skyrmion nucleation by magnetic force microscopy

Atomic and magnetic force microscopy (AFM and MFM) were conducted on CoGd films to investigate skyrmion nucleation. The main text presented MFM data for a 10 nm thick CoGd film, Pt/CoGd(10 nm)/W at room temperature in zero field, indicating the formation of magnetic structure and skyrmions. Here we present additional data obtained on a 8 nm thick CoGd film. The MFM figure below (Fig. S1(b)) shows the appearance of ~ 50 nm diameter magnetic contrast (in the lower right hand corner of the image) in a Pt/CoGd(8 nm)/W film at room temperature in zero-field, also suggesting the formation of a magnetic skyrmion. As with the MFM data in the main text, prior to imaging, the film was demagnetized with an AC in-plane magnetic field.

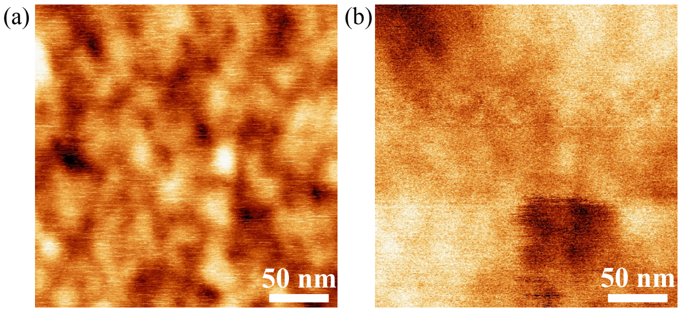

Figure S1: (a) AFM and (b) MFM imaging in Pt/CoGd(8 nm)/W that shows a 50-nm diameter contrast in the lower right hand corner of the image, suggesting the formation of a magnetic skyrmion. The surface roughness was about 0.2 nm (rms).

Below, we show similar skyrmion-like textures imaged in Pt/CoGd(15 nm)/W obtained in different areas.

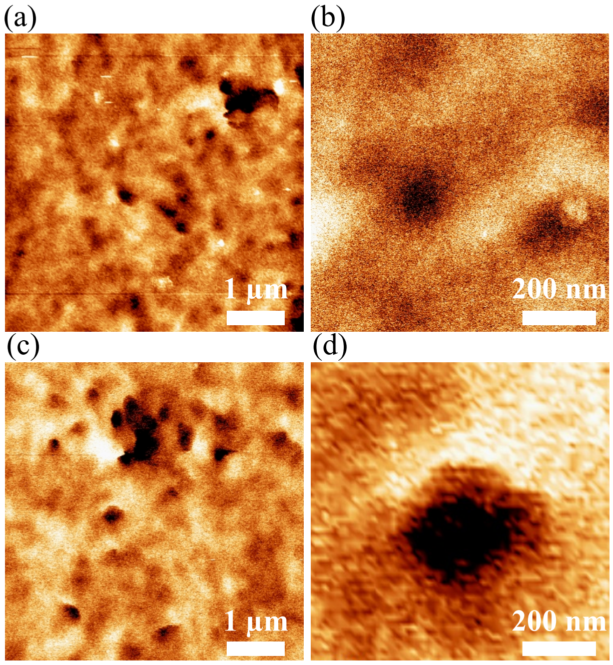

Figure S2: (a) and (c) Different areas were probed by MFM in Pt/CoGd(15 nm)/W and a finer scan was performed to evidence ~ 100 nm and ~ 200 nm skyrmion-like texture in (b) and (d), respectively.
